# Circadian rhythms of gut microbiota and plaque vulnerability: mechanisms and chrono-microbiota modulation interventions

**DOI:** 10.1080/19490976.2025.2532703

**Published:** 2025-07-12

**Authors:** Taiyu Zhai, Xueyang Zou, Zichen Zhang, Yifei Wang, Lin Shi, Wenbo Ren, Jing Huang

**Affiliations:** a Department of Clinical Laboratory, The First Hospital of Jilin University, Changchun, China; b College of Medical Technology, Beihua University, Jilin, China

**Keywords:** Stability of atherosclerotic plaques, circadian rhythm, gut microbiota, chrono-modulation, biological clock synchronization, host-microbe interaction

## Abstract

The stability of atherosclerotic plaques constitutes the fundamental pathological basis for acute cardiovascular events, and their circadian rhythm characteristics highlight the essential role of dynamic interactions between the host and microorganisms. This review systematically elucidates the multifaceted mechanisms by which disruptions in the circadian rhythm of the gut microbiota contribute to plaque destabilization. Specifically, the microbiota modulates endothelial function, immune homeostasis, and vascular inflammation via rhythmic variations in metabolites. Perturbations in this rhythm compromise the structural integrity of plaques through a synergistic “metabolic-immune-vascular” network. Furthermore, the review unveils the bidirectional regulation between the host’s circadian clock and the microbiota’s rhythm. Innovatively, we propose “Chronotherapy-based Microbiome Modulation (CMM),” a strategy that reestablishes synchrony between the host and microbiota rhythms through time-restricted feeding, time-specific probiotics, and drugs targeting the circadian clock, thereby, it is possible to improve plaque stability by regulating the host’s gut microbiota. The clinical translation of these findings requires overcoming technical challenges, such as personalized time window prediction and microbiota ecological risk assessment, and integrating multi-omics dynamic monitoring with AI modeling and optimization strategies. This review presents a novel perspective on the regulation of plaque stability.

## 1. Introduction

Atherosclerotic cardiovascular disease (ASCVD)-related acute events exhibit significant circadian rhythm characteristics, which are deeply associated with the human body’s circadian regulatory mechanisms. Clinical observations have found that cardiovascular events such as acute coronary syndrome, ischemic stroke, and malignant arrhythmias show a concentrated peak in the early morning, closely related to the circadian regulation of core physiological indicators such as blood pressure fluctuations, vascular endothelial function, and coagulation system activity.^1–3^ Notably, this chronobiological characteristic is not only reflected at the clinical symptom level but also in the underlying pathological mechanisms, with approximately 35–40% of acute coronary syndrome events occurring between 6:00 and 12:00, and the peak of coronary stent thrombosis occurring more precisely around 7:00.^4^ This time specificity suggests a need to break through traditional cognitive frameworks and delve deeper into the regulatory networks of biological rhythms.

Plaque rupture is one of the main pathological mechanisms of acute coronary syndrome, closely related to reduced plaque stability. Traditionally, the rhythmicity of acute coronary syndrome has been attributed to endogenous factors such as the natural increase in blood pressure and heart rate, increased sympathetic nerve activity, and the circadian rhythm of platelet activation.^1,5^ However, clinical studies have found that even after inhibiting sympathetic nerve activity through drugs (such as β-blockers), the early morning peak of cardiovascular events is only reduced by about 30%.^6^ This suggests that, besides the host’s own circadian clock, there may be other key pathological mechanisms driving the “time window” of plaque rupture. This unresolved clinical paradox prompts researchers to turn their attention to the exogenous regulatory network closely interacting with the host’s circadian clock: the gut microbiota. As the host’s second circadian clock, it also exhibits diurnal oscillation characteristics and has garnered attention in the field of cardiovascular disease research.^7,8^ The diurnal oscillation of the gut microbiota plays a crucial role in the host’s physiological processes, especially in maintaining the metabolic balance of nutrients.^9,10^ Metabolic imbalance is a fundamental feature of ASCVD.^11^

The symbiotic relationship between the host and gut microbiota extends far beyond traditional metabolic collaboration. The diurnal oscillation of the gut microbiota may drive changes in plaque stability through a trinity of “metabolism-immunity-vasculature” mechanisms. Studies have shown that the circadian rhythm of the gut microbiota is closely related to the host’s circadian clock.^12^ The host’s core circadian clock genes (such as BMAL1, CLOCK, PER/CRY) shape the periodic fluctuations in the structure and function of the gut microbiota by regulating feeding behavior, bile acid secretion, and intestinal motility rhythms.^13–15^ In turn, fluctuations in the microbiota can regulate the host’s metabolic rhythms and other peripheral circadian clocks, affecting lipid metabolism and glucose homeostasis, which may influence the stability of atherosclerotic plaques.^12,16,17^ Furthermore, metabolites of the gut microbiota, such as short-chain fatty acids and other microbial metabolites, can interact with the host’s immune system by activating G protein-coupled receptors (GPCRs) and inhibiting histone deacetylases, regulating inflammatory responses and the function of immune cells (such as macrophages), thereby affecting the inflammatory state of the vasculature and plaque stability.^18,19^ Disruptions in the host’s circadian clock can lead to microbial dysbiosis, triggering metabolic disorders and immune system imbalances.^20,21^ Studies have also found that the diurnal oscillation of the gut microbiota can further affect plaque stability by influencing the host’s endothelial function and vascular reactivity. For example, changes in the gut microbiota may lead to endothelial dysfunction and increased vascular stiffness, which are risk factors for atherosclerotic plaque rupture.^22,23^ These dynamic interactions between the gut microbiota and the host suggest that the gut microbiota is not only the “chemical plant” of host metabolism but may also be the “external pacemaker” regulating the host’s chronobiology, although the specific mechanisms still need further elucidation.

This review is the first to elucidate the multi-layered mechanisms by which disruptions in the diurnal rhythm of the gut microbiota drive plaque destabilization and proposes the concept of “chrono-microbiota modulation (CMM),” aiming to provide a novel paradigm for the prevention and treatment of ASCVD. The article starts from the regulatory network of the host/environment on microbiota rhythms, analyzes the mechanisms of plaque destabilization mediated by the metabolism-immunity axis, and further explores novel therapeutic strategies based on chronobiology and their clinical translation challenges.

## 2. Symbiotic regulation between the host’s circadian rhythm and the rhythmicity of gut microbiota

The symbiotic regulation between the host circadian clock and gut microbiota rhythms constitutes an important foundation for maintaining health in organisms. As the core system regulating metabolism, immunity, and cardiovascular function, the host’s circadian rhythm is coordinated by the central clock in the suprachiasmatic nucleus of the hypothalamus and peripheral clocks distributed throughout the body.^24^ Disruptions in this system are closely associated with metabolic disorders and cardiovascular diseases.^25^ Notably, the gut microbiota, as a crucial peripheral clock, forms a unique bidirectional regulatory network with the host circadian clock. Studies have shown that host circadian clock genes have a decisive influence on the microbiota rhythms: Bmal1-deficient mice and mice simulating shift work not only exhibit gastrointestinal peripheral clock disruptions but also significantly reduced microbiota rhythmicity in short-chain fatty acid fermentation and lipid metabolism.^26^ This regulatory relationship is also dynamically influenced by external environmental signals, such as light signals, which can indirectly affect microbiota homeostasis and host defense mechanisms by regulating the circadian rhythm of intestinal innate lymphoid cells (ILC3s), revealing the pivotal role of environmental factors in host-microbe interactions.^27^

Further research has revealed that the gut microbiota is not only a passive responder to host physiological processes but also an active regulator. The diurnal fluctuations in its composition, spatial distribution, and metabolic output maintain barrier function, regulate mucosal immunity, and stabilize immune cell populations, thereby serving as an important guarantee for intestinal health.^20^ Once this dynamic balance is disrupted by jet lag or shift work, it leads to microbiota dysregulation and triggers metabolic disorders such as glucose intolerance and obesity, highlighting the crucial value of microbial rhythms to host health.^16^ Notably, microbial rhythm disruptions primarily regulate the host metabolic network through non-light signals such as diet, and their mechanisms of action depend on both circadian clock genes and also affect metabolic targets through independent pathways.^7^ In this process, feeding time, as a core regulator of the circadian rhythm, can significantly amplify the diurnal fluctuations in microbiota metabolic activities through strategies such as intermittent fasting, thereby regulating the host’s central and peripheral clocks via microbial metabolites, providing novel intervention ideas for metabolic diseases such as atherosclerosis.^28^

The complex relationship between the host circadian clock and gut microbiota is further reflected in the regulation of host epigenetic pathways, particularly histone modifications. Studies have shown that the gut microbiota drives diurnal rhythmic changes in metabolism-related genes by regulating the expression of histone deacetylase 3 (HDAC3) in the small intestinal epithelium.^16^ This microbe-dependent HDAC3 rhythmic expression not only synchronizes histone acetylation levels but also dynamically regulates metabolic gene transcription activity and nutrient absorption efficiency, thereby establishing the molecular basis for microbe-host metabolic coordination. Additionally, the microbiota can affect the composition of the intestinal circadian transcriptome and stabilize the diurnal rhythm of the intestine. The gut microbiota significantly influences host energy metabolism through the circadian rhythm transcription factor NFIL3, which is regulated by the microbiota through the diurnal rhythm clock in epithelial cells.^29^ This underscores the intricate relationship between the host circadian clock and gut microbiota rhythms. The regulation of the host rhythmic system by timed feeding and microbiota depletion exhibits both synergistic and independent characteristics, suggesting that dietary rhythm and microbiota homeostasis jointly constitute the driving system of the host circadian clock.^30^ Furthermore, they affect host physiology through metabolites and structural components, thereby regulating the host’s circadian rhythm.^31^ This interaction has important implications for host immune and metabolic functions. Understanding these mechanisms can enhance our understanding of circadian rhythm disorders and potential therapeutic approaches.

Overall, the bidirectional interaction between the host circadian rhythm and gut microbiota emphasizes the importance of maintaining synchronized circadian clocks for optimal health. Maintaining this dynamic balance is crucial for preventing metabolic diseases, cardiovascular abnormalities, and other modern high-incidence diseases. With the deep intersection of chronobiology and microbiomics, time-based therapies and precision dietary interventions based on gut microbiota rhythm regulation may become innovative breakthroughs in reshaping circadian clock synchrony and achieving disease prevention.^12^

## 3. Mechanisms underlying the destabilization of plaques driven by disturbances in the diurnal rhythm of gut microbiota

**Figure 1.**
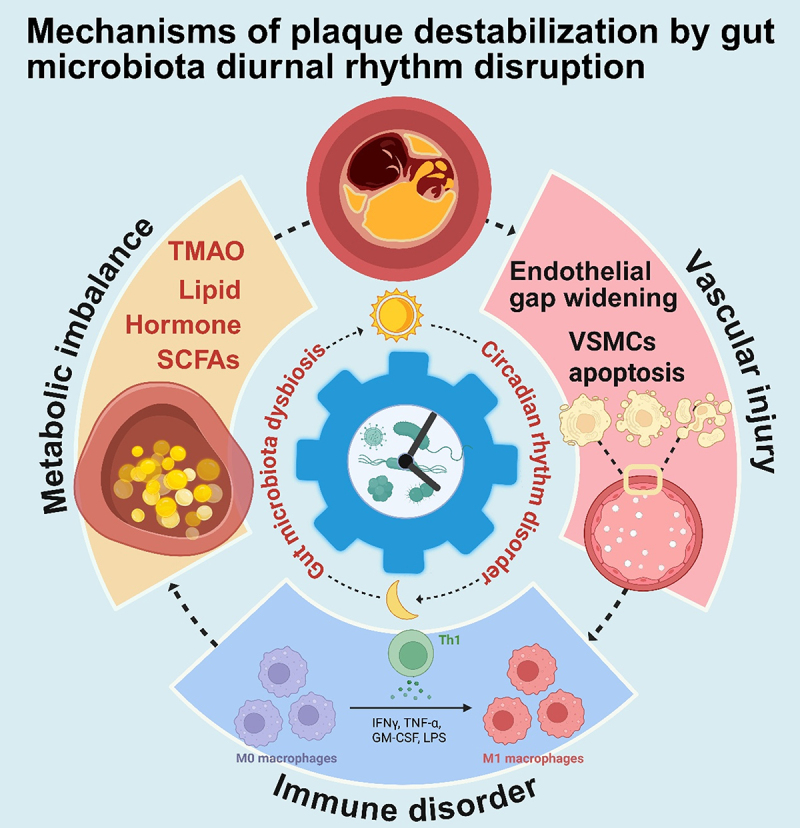
Mechanisms of plaque destabilization by gut microbiota diurnal rhythm disruption.

Research into the mechanisms by which disruptions in the circadian rhythm of gut microbiota drive plaque instability is reshaping our understanding of the pathological process of atherosclerosis. Traditional theories attribute plaque stability to the synergistic action of three core mechanisms: metabolic regulation, immune response, and vascular function.^32,33^ However, recent evidence suggests that circadian fluctuations in gut microbiota integrate this “metabolism-immune-vascular” trinity system to dynamically regulate plaque stability. Specifically, gut microbiota not only exhibit diurnal periodic fluctuations in quantity and composition, but their colonization sites also migrate during the day, and their metabolic functions demonstrate significant temporal differentiation: core functions such as DNA repair and energy metabolism are active at night, while functions such as detoxification, motility, and environmental sensing exhibit diurnal advantages.^34,35^ This precise circadian regulation is highly dependent on the host’s feeding rhythm, and disordered eating can lead to phase shifts in microbial function, causing temporal mismatches in metabolite production and immune regulation. Notably, the rhythm interaction between the microbiota and host is bidirectional – the circadian activity of microorganisms regulates the host’s physiological state through metabolites and immune signals, while metabolic disorders in the host can exacerbate microbiota dysbiosis in turn, thereby forming a vicious cycle of “metabolic imbalance-immune disorder-vascular damage.”^7,12^ In this process, disrupted microbiota rhythms threaten plaque stability through multiple pathways: on the one hand, deregulated short-chain fatty acid (SCFAs) metabolism leads to endothelial dysfunction and abnormal lipid deposition; on the other hand, diurnal immune dysregulation exacerbates plaque inflammation, ultimately driving the plaque toward an unstable state (Figure 1). This mechanism groundbreakingly incorporates the circadian rhythm of microorganisms into the comprehensive regulatory network of plaque stability, not only revealing a novel circadian regulatory axis for the occurrence of cardiovascular diseases but also suggesting that synchronizing “host feeding rhythm-microbial function phase” may become an innovative intervention strategy for stabilizing atherosclerotic plaques and preventing acute cardiovascular events.

### 3.1. The impact of metabolic imbalance driven by circadian rhythm disruption in gut microbiota on plaque stability

The mechanisms linking metabolic imbalance driven by circadian rhythm disruption in gut microbiota to plaque stability reveal the spatio-temporal dynamic characteristics of microbe-host interactions in cardiovascular pathology. As the core linkage between the two, the rhythmic fluctuations of microbial metabolites profoundly affect the progression of atherosclerosis by directly regulating vascular endothelial function and systemic metabolic homeostasis. Studies have shown that circadian rhythm disruption in gut microbiota not only disrupts microbial homeostasis but also triggers systemic metabolic disorders in the host, with a significant association between this imbalance and increased cardiovascular risk identified a decade ago.^36^ In recent years, with in-depth research on key metabolites such as trimethylamine-N-oxide (TMAO) and SCFAs, the “positive-negative balance” mechanism by which they regulate plaque stability has gradually become clear.^37–39^

Among them, TMAO, a typical product of gut microbiota metabolism, is closely related to plaque instability when its levels rise.^40^ Circadian rhythm disruption in gut microbiota can promote TMAO production by altering bile acid and lipid metabolism pathways, thereby exacerbating metabolic disorders and increasing cardiovascular risk.^41–43^ TMAO destabilizes plaques through multiple mechanisms: firstly, it induces pyroptosis in vascular endothelial cells, directly damaging endothelial barrier function^44^; secondly, it inhibits antioxidant signaling pathways such as Nrf2/ABCA1, exacerbating oxidative stress and promoting lipid accumulation in foam cells^45^; thirdly, it enhances platelet reactivity and expression of tissue factor in vascular endothelial tissue, increasing the risk of thrombosis, which is also one of the important factors leading to plaque rupture^46^; additionally, TMAO activates inflammatory signaling pathways, driving infiltration and activation of inflammatory cells within plaques, further weakening plaque structure.^47,48^ Notably, reducing TMAO levels may improve plaque microenvironment stability by promoting M2 polarization of macrophages and enhancing the clearance capacity of apoptotic cells.^49^ These findings not only reveal the core pathological role of TMAO in atherosclerosis but also provide potential targets for intervening in plaque progression by regulating microbial metabolites.

Circadian rhythm disruption in gut microbiota also interferes with the production and release of SCFAs (including acetate, propionate, and butyrate), further affecting the stability of atherosclerotic plaques.^50^ As key products of gut microbiota metabolism, SCFAs inhibit the progression of atherosclerosis through multiple mechanisms such as regulating lipid metabolism, maintaining endothelial function, and modulating immune responses. Specifically, SCFAs can improve lipid metabolism abnormalities, for example, by regulating the expression of lipid metabolism-related genes to reduce lipid deposition in the vascular wall, while maintaining the integrity of vascular endothelium. It has been verified that butyrate regulates the phosphorylation level of vascular endothelial cadherin (VEC) by activating FFAR2/FFAR3 receptors and the c-Src kinase signaling pathway, thereby reducing endothelial permeability and enhancing barrier function.^51^ Furthermore, SCFAs can interfere with the transduction of proinflammatory signaling pathways by altering the composition of cellular membrane lipid rafts, reducing local vascular inflammation.^52^ Their anti-inflammatory effects are further manifested by activating G protein-coupled receptors and inhibiting histone deacetylases (HDACs), significantly reducing oxidative stress levels and inhibiting the proinflammatory polarization of macrophages, while promoting the function of regulatory T cells, jointly maintaining the stability of the plaque microenvironment.^53–55^ Clinical studies have also found that SCFA levels are closely related to individual lipid profiles and the progression of carotid atherosclerosis, suggesting their potential as novel biomarkers for assessing plaque stability and cardiovascular risk.^56^ These findings emphasize the potential of SCFAs as therapeutic agents for preventing plaque rupture and managing cardiovascular diseases. Additionally, metabolites such as urolithin A (UA) and protocatechuic acid (PCA) exhibit unique vascular protective potential: UA enhances endothelial function by improving gut microbiota diversity, while PCA inhibits diabetic vascular inflammation through the Akt/eNOS pathway.^57,58^ These findings expand the molecular landscape of microbial metabolite regulation of cardiovascular health.

This metabolic regulatory network essentially reflects the dynamic dialogue between the host and microorganisms over time. Circadian rhythm disruption in gut microbiota disrupts the phase coordination of bile acid metabolism, lipid homeostasis, and immune rhythms, forming a vicious cycle of “metabolic imbalance-uncontrolled inflammation-endothelial damage.” For example, functional phase shifts in gut microbiota caused by sleep disorders can disrupt the balance between TMAO and SCFAs, accelerating plaque destabilization through oxidative stress and endothelial pyro ptosis; whereas metabolites such as UA demonstrate “systemic repair” advantages through multi-target regulation. This spatio-temporal interaction mechanism suggests that future intervention strategies need to break through the traditional single-target model and shift to an overall perspective of “rhythm resynchronization” - restoring the coupling of metabolic oscillations with host rhythms through time-dependent dietary interventions or photoperiod regulation may more effectively block the pathological process.

### 3.2. Disruption of circadian rhythm in gut microbiota induces immune imbalance in the host, affecting plaque stability

Atherosclerosis, a chronic inflammatory disease, exhibits variations in plaque stability that are invariably accompanied by the profound involvement of the immune system.^59,60^ Studies have demonstrated that the dynamic infiltration and functional polarization of immune cells are pivotal factors determining plaque stability. Innate immune cells (such as neutrophils, monocytes, and macrophages) directly disrupt plaque structure through the release of inflammatory mediators and proteases^61–65^; whereas adaptive immune cells (such as Th1/Th17 cells and regulatory T cells) regulate the balance of the plaque microenvironment via the interplay of pro-inflammatory and anti-inflammatory signals.^66–68^ Especially during plaque progression, the interaction between macrophages and vascular smooth muscle cells becomes the focal point. Matrix metalloproteinases (MMPs) secreted by macrophages degrade the collagen in the fibrous cap, while the imbalance between the proliferation and apoptosis of smooth muscle cells impairs plaque repair capacity. The synergistic dysregulation of these two factors ultimately results in the transformation of plaques from stable to vulnerable.^69–71^ Thus, the transition in plaque stability in atherosclerosis is fundamentally the consequence of the combined effects of local immune microenvironment imbalance and systemic immune regulatory dysfunction.

Recent research has unveiled that the gut microbiota plays a pivotal role in systemic immune regulation through a metabolite-mediated “gut-immune axis.” The gut microbiota periodically secretes signaling molecules, including short-chain fatty acids (e.g., butyrate, propionate), tryptophan metabolites, and bile acids. These metabolites directly regulate the differentiation and function of immune cells through receptor binding or epigenetic modifications, thereby maintaining both local and systemic immune homeostasis.^72,73^ For instance, short-chain fatty acids upregulate Foxp3 gene expression by inhibiting histone deacetylases, thereby promoting the generation of regulatory T cells (Treg) to enhance immune tolerance^74^; bile acid metabolites inhibit the activity of pro-inflammatory Th17 cells by modulating the Th17/Treg cell balance.^75^ Furthermore, these metabolites also act on dendritic cells and macrophages, reducing the release of pro-inflammatory factors, thereby creating an immunosuppressive microenvironment in the gut and distant organs (such as atherosclerotic plaques).^76,77^ Notably, disruption of the circadian rhythm in gut microbiota leads to abnormal secretion patterns of metabolites, disrupting systemic immune homeostasis through epigenetic reprogramming or direct interference with immune cell function. This mechanism offers a novel perspective for elucidating the progression of chronic inflammatory diseases, such as atherosclerosis.^78^ Based on these findings, targeting the metabolic activities of the gut microbiota (such as supplementing specific metabolites or disrupting microbiota rhythms) has emerged as a potential therapeutic strategy. By restoring the Th17/Treg balance or reestablishing rhythmic secretion of metabolites, it is anticipated that novel intervention pathways for autoimmune and inflammatory diseases will be identified.^79–81^

Based on existing evidence, targeting the metabolic activities of the gut microbiota (such as supplementing specific metabolites or disrupting microbiota rhythms) may emerge as a novel strategy to stabilize plaques by restoring immune balance or reestablishing rhythmic secretion of metabolites. Meanwhile, these findings closely associate the spatiotemporal rhythmic characteristics of gut microbiota with host immune regulation, suggesting that the next phase of research should focus on how circadian oscillations in the microbiota reshape plaque microenvironment stability through metabolite-immune crosstalk. This will establish a theoretical foundation for developing more precise intervention methods.

### 3.3. Disruption of circadian rhythm in gut microbiota regulates vascular function and affects plaque stability

The regulatory mechanism by which circadian rhythm disruption in gut microbiota influences vascular function and plaque stability unveils a dynamic interaction network between the microbiome and the cardiovascular system. As a pivotal aspect of the atherosclerotic process, vascular endothelial dysfunction not only maintains vascular homeostasis by regulating tone, inhibiting inflammation, and preventing thrombosis, but its impairment directly precipitates plaque formation and substantially elevates the risk of rupture.^82–84^ Recent studies have demonstrated that disruptions in the circadian rhythm of gut microbiota can exacerbate this pathological process via metabolite-immune interactions. When the diurnal oscillation of gut microbiota is disturbed, the synthesis of key protective metabolites, such as indolepropionic acid (IPA), is markedly decreased, resulting in the malfunction of regulatory mechanisms for plaque stability. In physiological conditions, IPA preserves the structural integrity of the fibrous cap by inhibiting abnormal activation of MMP-9. Experimental evidence has shown that it concurrently reduces MMP-9 activity and levels of oxidative damage markers in oxidative stress models, thereby enhancing the mechanical stability of plaques.^85–87^ Additionally, this metabolite facilitates cholesterol efflux in macrophages by activating the miR-142-5p/ABCA1 signaling axis, inhibiting foam cell formation, and consequently improving endothelial function and augmenting the biological stability of plaques.^88,89^ However, metabolic imbalances resulting from disruptions in gut microbiota circadian rhythms will directly impair the production of such protective compounds, leading to decreased endothelial repair capacity and heightened risk of plaque rupture.^90^

More importantly, the disruption of circadian rhythm in gut microbiota exhibits systemic characteristics in disrupting vascular homeostasis.^12,29^ This imbalance not only initiates a cascade of reactions through the “gut-immune-vascular” axis but also compromises intestinal barrier function with a disrupted gut microbiota structure, fostering pathogen translocation and inciting systemic inflammation.^91–93^ It further alters immune cell function through metabolic reprogramming, cultivating a chronic low-grade inflammatory milieu. Further research has revealed that disruptions in gut microbiota can directly interfere with mitochondrial energy metabolism in endothelial cells, disrupting tight junctions between cells, resulting in increased endothelial permeability and decreased repair capacity.^94^ When coupled with metabolic syndromes or diabetes, this detrimental effect is substantially amplified, forming a complex pathological network that underscores the crucial role of gut microbiota circadian rhythm regulation in cardiovascular diseases.^42,95^

In summary, the circadian rhythm regulation of gut microbiota has emerged as a novel target for the prevention and treatment of atherosclerosis. Existing research has delineated a multidimensional framework of action: the gut microbiota circadian rhythm dynamically modulates endothelial function and plaque stability through metabolites, while also influencing systemic inflammatory states via immune regulatory networks. This spatiotemporal interaction offers a novel perspective for the prevention and treatment of cardiovascular diseases. However, the specific molecular mechanisms of gut microbiota oscillatory signal transduction, the dose-response relationship of key metabolites, and the temporal and spatial regulation patterns remain to be further elucidated. Future research should integrate microbiome, metabolome, and single-cell technologies to systematically decipher the molecular map of the synergistic regulation of vascular homeostasis by the “gut microbiota circadian rhythm-host circadian rhythm.” This will establish a theoretical foundation for the development of precise intervention strategies based on gut microbiota circadian rhythm regulation and pioneer a new chronobiology-based treatment paradigm for the prevention and treatment of atherosclerosis.

## 4. Chrono-microbiota modulation interventions

CMM provides a novel and dynamic approach for the prevention and treatment of atherosclerosis by precisely coordinating the circadian rhythms of the host and its gut microbiota. In contrast to the static model of traditional microbiota modulation, CMM emphasizes the temporal synchronization between the physiological rhythms of the host and the fluctuations in microbiota function. Its core mechanism arises from the host’s circadian clock shaping the structure and function of the gut microbiota through dietary rhythms, body temperature variations, and hormone secretion (such as cortisol and melatonin), which subsequently influences plaque stability via a bidirectional regulatory network.^7,28,42,96^ For instance, the colonization efficiency of lactobacillus supplements is 30% higher during the host’s active period (night) than during the rest period, owing to the circadian phase dependency of intestinal epithelial mucus secretion and immune tolerance.^97,98^ Similarly, the timing of time-restricted feeding (TRF), an important strategy for enhancing metabolic health, optimizes metabolic responses by synchronizing circadian gene expression, but its benefits are highly contingent upon the phase alignment between the feeding window and the host’s circadian clock.^99–101^ Morning TRF (6:00–15:00) is more effective than afternoon TRF (11:00–20:00) in restoring microbiota metabolic rhythms and augmenting microbial diversity, with mechanisms involving HDAC inhibition-mediated reduction of plaque inflammatory cell infiltration.^102,103^ Additionally, the gut microbiota serves as a crucial regulator of the rhythmicity of the hypothalamic-pituitary-adrenal (HPA) axis. Its rhythmic nature is vital for maintaining the normal circadian rhythm of glucocorticoid secretion.^104^ Glucocorticoids play a pivotal role in regulating stress responses and maintaining plaque stability.^105^ Any disruption to their secretory rhythms can lead to various pathophysiological alterations. Further research suggests that Lactobacillus reuteri may be a key bacterial strain mediating the regulatory effects of the gut microbiota on the rhythmicity of the HPA axis.^104^ However, it is worth noting that relevant studies often lack sufficient disclosure regarding sample collection times. Given the inherent rhythmicity of the gut microbiota and the significant impact of sampling time on sequencing outcomes, it is essential to approach the interpretation of similar research findings with caution.^9^ Nevertheless, the aforementioned findings convincingly demonstrate that the gut microbiota regulates stress responsiveness in a circadian manner, which is crucial for the organism’s ability to adaptively respond to stressors at different times throughout the day. These discoveries further highlight the central importance of “temporal precision” in the design of intervention strategies, emphasizing the need to fully consider the influences of the host’s circadian clock and the rhythmicity of the gut microbiota when formulating and implementing microbiota-based interventions to maximize their effectiveness.

At the molecular level, the host’s BMAL1/CLOCK complex influences short-chain fatty acid (SCFAs) synthesis by regulating microbiota genes such as butyrate kinase (buk), whereas microbiota metabolites like butyrate regulate the host’s circadian clock genes through PPARγ signaling, thereby forming a bidirectional rhythmic circuit.^36,106^ This interaction extends to metabolic-immune regulation: SCFAs peak during the host’s active period, inhibiting the NF-κB pathway in endothelial cells through GPR41/43 receptors, with time-dependent anti-inflammatory effects intimately linked to plaque stability.^107–109^ Conversely, TMAO synthesis is governed by the circadian activity of the microbiota’s FMO3 enzyme, and restricting choline intake in the morning can substantially decrease its production levels, furnishing a rationale for time-specific dietary interventions.^110,111^ Notably, the effects of specific probiotics on bolstering plaque stability by reducing intestinal permeability and serum LPS concentrations exhibit diurnal variations, indicating that microbiota interventions must dynamically adapt to the host’s immune rhythms.^112^

Based on these mechanisms, CMM focuses on three primary practical directions: firstly, rhythmic coordination through TRF, which synchronizes the eating window with the host’s circadian clock to improve blood glucose and lipids while modulating matrix metalloproteinase (MMPs) activity and enhancing plaque mechanical stability^113–115^; secondly, time-specific interventions with probiotics/prebiotics, wherein, for example, bifidobacterium breve is efficacious only when administered prior to disease onset in colitis mouse models,^116^ whereas the prebiotic inulin, by promoting the proliferation of Roseburia, can both ameliorate diabetic metabolic disorders and diminish plaque necrosis core area^117,118^; thirdly, the development of circadian clock-targeting drugs, such as the REV-ERBα agonist SR9009, which improves metabolic disorders and dysbiosis by synchronizing host circadian clock genes.^119^ Clinical studies have confirmed that TRF shifts the gut microbiota structure toward anti-inflammatory phyla like Bacteroidetes and Actinobacteria, reducing plaque inflammatory infiltration through HDAC inhibition,^120^ while the metabolic benefits of probiotic supplementation in obese adolescents are significantly correlated with the timing of administration.^119,121^

Current research has initially established a three-dimensional intervention network of “rhythm synchronization-metabolic regulation-immune homeostasis,” transitioning cardiovascular disease prevention and treatment from static regulation of single targets to precise temporal and spatial dynamic interventions. Future research endeavors need to further delineate the molecular nuances of host-microbiota rhythmic interactions, optimize timing window configurations and individual adaptations, providing theoretical backing for the chronobiological treatment of chronic diseases such as atherosclerosis.

## 5. Key differences between circadian microbiome modulation and traditional approaches to cardiovascular disease treatment

The core distinction between CMM and traditional ASCVD treatment strategies resides in the dynamic integration of the circadian rhythm characteristics of both the host and the microbiota by CMM, rather than relying on static interventions targeting a single molecular target. Current mainstream treatments, such as statins, reduce LDL-C levels by continuously inhibiting HMG-CoA reductase activity, whereas antiplatelet agents like aspirin irreversibly block COX-1 activity to prevent thrombosis. Although these approaches effectively control disease progression, they neglect the circadian rhythm features of cardiovascular events, potentially leading to drug resistance or adverse effects due to their sustained action.^122,123^ For instance, statin-induced myotoxicity is attributed to its persistent inhibition of mitochondrial function, while the risk of gastrointestinal bleeding associated with aspirin use is linked to its 24-hour antiplatelet effect.^124,125^

**Figure 2.**
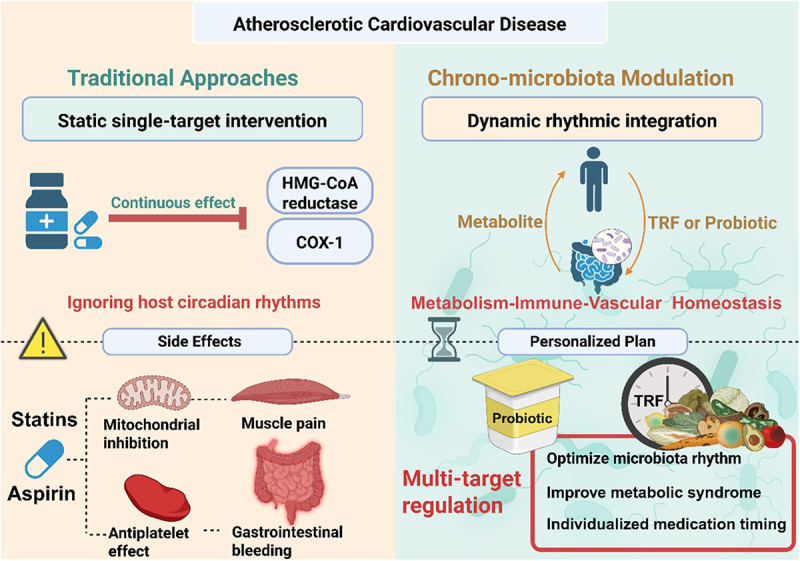
The key differences and advantages of circadian microbiome modulation over traditional approaches.

In contrast, the innovation of CMM lies in harmonizing the host-microbiota rhythm network through time-dependent interventions, thereby reestablishing the dynamic equilibrium of the plaque microenvironment. The biological foundation of this strategy stems from the circadian coordination of metabolic and immune functions between the host and the microbiota.^16^ The host’s biological clock modulates the composition and functional oscillations of the microbiota through mechanisms such as feeding behavior and the rhythmic secretion of bile acids. Conversely, microbiota metabolites reciprocally regulate the host’s peripheral biological clock, forming a bidirectional synchronization network.^7,12,21^ It has been demonstrated that TRF can optimize host energy utilization and mitigate metabolic syndrome by restoring the circadian rhythm of the gut microbiota.^115^ The temporal dependency of probiotic interventions is rooted in circadian physiological characteristics. For example, gastric acid pH peaks at night, which can diminish probiotic survival rates, providing a rationale for timed drug administration (Figure 2). However, the optimal timing for specific probiotic strains still necessitates individualized investigation.^126,127^

The translational paradigm of CMM can leverage the mature experiences of chronochemotherapy for tumors. For instance, adjusting the dosing time of oxaliplatin can modulate its pharmacokinetic profile in the body, thereby influencing its efficacy and toxicity.^128^ Continuous infusion of 5-Fluorouracil combined with rhythmic modulation can augment its therapeutic efficacy.^129,130^ These tumor treatment regimens offer methodological insights for the clinical translation of CMM. CMM can optimize the phase alignment of microbiota metabolites with host receptor activity through a comparable temporal logic.

Furthermore, the multi-target regulatory attributes of CMM enable it to concurrently act on the metabolic-immune-vascular axis. The microbiota metabolite butyrate not only suppresses the NF-κB inflammatory pathway by activating GPR43 but also upregulates vascular endothelial cadherin expression by inhibiting HDACs, thereby enhancing endothelial barrier function.^51,131,132^ Such systemic regulation is challenging to achieve with single-target drugs. In the future, the clinical translation of CMM will necessitate integrating personalized rhythm typing (such as based on salivary cortisol rhythm or core body temperature monitoring) with the development of intelligent drug delivery systems to surmount the spatiotemporal constraints of traditional therapies.

## 6. Clinical translation challenges and future directions

The clinical application of time-sensitive microbiota interventions in regulating plaque stability is confronted with intricate challenges stemming from individual heterogeneity and technological bottlenecks. Despite demonstrating potential through regulating the host’s circadian clock and metabolic balance, the core challenge in translation lies in the precise prediction of individualized time windows. Significant individual differences exist in the host’s circadian phase, microbiota composition, and metabolite rhythms. For instance, the richness and stability of gut microbiota in obese individuals are significantly lower than those in healthy individuals,^111,133^ rendering unified intervention protocols (such as fixed morning time-restricted feeding) potentially ineffective or even harmful for some patients. Currently, individualized prediction systems need to integrate multidimensional rhythmic parameters: the diurnal slope of salivary cortisol reflects hypothalamic-pituitary-adrenal axis rhythms,^134^ the nadir of core body temperature marks the host’s core circadian phase,^135^ and the peak phase of activity of microbiota metabolic enzymes such as CutC correlates with the synthesis rhythm of TMA, which has been shown to regulate the host’s circadian clock.^136^ However, it should be emphasized that a single indicator is insufficient to fully characterize the host-microbiota rhythmic features, necessitating the construction of a comprehensive assessment model through multi-omics dynamic data. One of the core values of constructing such a comprehensive personalized microbial rhythm assessment model lies in its potential to predict plaque vulnerability. Specific microbial rhythm characteristics, such as shifts in the peak phase of key metabolic enzyme activities, attenuation or disruption of the circadian oscillation amplitudes of core beneficial/harmful bacterial abundances, and abnormal rhythmic fluctuation patterns of specific metabolites, may serve as early warning biomarkers. These biomarkers reflect the spatiotemporal dysregulation state of host-microbe interactions, which has been demonstrated to be closely associated with elevated levels of vascular inflammation, endothelial dysfunction, and ultimately, plaque instability.^19,22,44,86^ Therefore, accurately delineating personalized microbial rhythm profiles is not merely for determining the optimal intervention time windows; it itself may emerge as a novel method for assessing cardiovascular risk and predicting dynamic changes in plaque stability.

**Figure 3.**
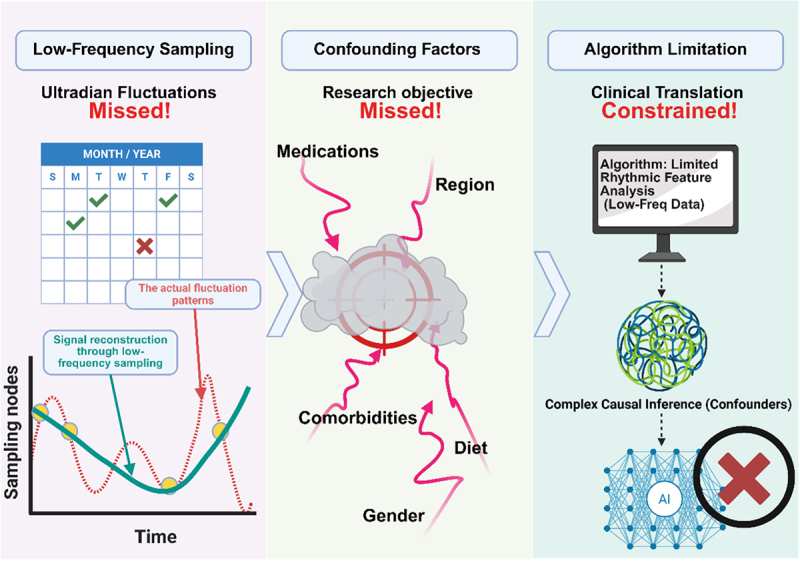
Triple technical barriers impeding the causal chain from microbiota rhythms to plaque stability: low-frequency sampling obscures ultradian signals (left), confounders disrupt association validation (center), and analytical constraints hinder clinical translation (right).

This process faces triple technical barriers (Figure 3): firstly, the low-frequency sampling in existing studies (1–2 times per day) is prone to missing the ultradian fluctuation signals of microbiota and metabolites; Secondly, confounding factors such as drug interference and comorbidity status affect the causal association verification between microbiota rhythms and plaque stability, necessitating Mendelian randomization or interventional studies to elucidate mechanisms. More crucially, low-frequency data limit the algorithm’s ability to analyze rhythmic features, while confounding factors increase the complexity of causal inference, further constraining its clinical translation. Specifically, widely used medications, such as proton pump inhibitors (which significantly alter the gastric acid environment and, consequently, the downstream microbiota),^137^ antibiotics (which directly disrupt microbiota composition and function),^138^ and statins (which affect cholesterol metabolism and potentially influence microbiota metabolism),^139^ can all substantially interfere with the rhythmic host-microbiota interactions and the underlying mechanisms by which they impact the vasculature. Similarly, dietary habits – including the macronutrient ratio, meal timing patterns, and specific dietary components are key drivers that shape the composition, abundance, metabolic activity, and circadian rhythms of the gut microbiome.^140,141^ If these confounding variables are not adequately documented, standardized, or statistically controlled in studies, they will severely impede the analysis of the core effects of microbiota rhythm interventions and obscure the true causal relationships between these interventions and plaque stability. Consequently, this makes it difficult to generalize findings from observational studies and complicates the interpretation of results from clinical trials.

When discussing the translational medicine risks associated with time-sensitive microbial interventions, and based on the current understanding within the field of microbial circadian rhythm research, we believe it is crucial to emphasize the following speculative viewpoints: Firstly, artificial manipulation of the microbial biological clock may entail cascading risks similar to an “ecological domino effect.” Notably, diet itself serves as a core and modifiable confounding variable that can be strategically employed as a relatively safe “chrono nutrition” intervention. By adjusting the timing of specific nutrient intake (rather than directly manipulating the microbiota), it may be possible to align with the host’s biological clock to some extent, thereby indirectly reshaping beneficial microbial rhythms. This approach also helps avoid the potential “ecological domino effect” risks associated with direct microbial interventions. However, the effectiveness of such dietary timing interventions is highly contingent on individual factors (such as baseline microbiota composition, host metabolic status, and dietary habits) and necessitates consideration of how regional and cultural differences might influence dietary adherence. Given that the mechanisms underlying microbial community dynamic equilibrium have not yet been fully elucidated by existing intervention methods, forced rhythmic synchronization could disrupt the compensatory feedback mechanisms among symbiotic microorganisms. This imbalance might persistently affect host physiological functions through a “metabolic memory effect.”^142^

Secondly, the intervention thresholds for the fluctuation amplitudes of specific metabolites need to be reevaluated. Taking TMAO as an example, its circadian fluctuations may engage in bidirectional regulation with bile acid synthesis pathways via the enterohepatic circulation. While enhancing clearance efficiency, this process might simultaneously alter the phase characteristics of cholesterol metabolism. Such cross-system temporal misalignment could potentially be an underlying trigger for metabolic liver injury.^143^ These inferences suggest that the clinical translation of microbial rhythm interventions requires the establishment of an “ecological buffer zone” concept. This involves maintaining the resilience of the microbial network while achieving rhythmic remodeling, and utilizing dynamic monitoring of temporal changes in beta diversity to preemptively detect ecological imbalance risks. It is crucial to emphasize that the early detection of personalized microbial rhythm disruptions and the precise forecasting of plaque vulnerability risks serve as the prerequisites and cornerstones for executing timely, accurate, and low-risk interventions within suitable time frames. Therefore, future studies are warranted to investigate the circadian dynamic release patterns of microbial metabolites and their effects on host physiology (e.g., plaque stability) at specific time points.

To systematically assess safety, future research needs to establish a multi-level validation system: firstly, conduct long-term follow-up studies in populations with circadian clock genotyping, combined with dynamic assessment of intervention timeliness using imaging biomarkers (Such as key indicators for assessing plaque vulnerability, including changes in the volume of the plaque lipid core, thickness of the fibrous cap, and the presence of intraplaque hemorrhage) can be used to dynamically evaluate the timeliness of interventions. And, we can explore the effectiveness of personalized microbial rhythm characteristics (as outputs from the model) in predicting the dynamic changes in these vulnerability indicators; secondly, adopt a multi-center randomized controlled trial (RCT) design, incorporating adaptive intervention strategies to dynamically adjust intervention protocols based on individual rhythmic features, avoiding ecological risks associated with a “one-size-fits-all” approach. Prospectively, it is advisable to include diverse population samples characterized by varying dietary habits, lifestyle backgrounds, and geographical origins. This will enable the evaluation of the generalizability and robustness of the interventions across different environmental settings; in addition, integrate novel monitoring technologies to enhance safety early warning capabilities. Ingestible electronic capsules can real-time track the concentrations of local intestinal metabolites (such as butyrate, TMAO) and microbiota activity, combined with deep learning models (such as Long Short-Term Memory networks) for integrated analysis of multi-omics time-series data, enabling dynamic prediction of optimal individual intervention time windows, and simultaneously evaluate the short-term risk score for plaque vulnerability based on microbial rhythm characteristics. Notably, although existing AI model prediction accuracy performs well in training sets, their external validation results still lack support from large-scale clinical data, requiring future research to establish cross-regional, cross-ethnic validation cohorts to improve model generalization. Significant disparities are observed in the core microbiota composition, dominant microbial species, and microbial metabolic functions across populations from different geographical regions. These disparities are collectively influenced by a multitude of factors, such as long-term dietary patterns, environmental exposures, and genetic backgrounds.^144^ Predictive models or intervention strategies developed without accounting for these regional differences may face significant challenges when implemented across diverse geographical areas. Consequently, establishing validation cohorts with extensive geographical representation is paramount for ensuring the global applicability of research outcomes.

## 7. Conclusion

Research on the rhythmic interaction between the host’s circadian clock and gut microbiota is driving the paradigm shift in atherosclerosis prevention and treatment from molecular targeted therapy to spatio-temporal dynamic regulation. Existing evidence reveals that microbiota, through diurnal coordination of metabolism-immunity-vascular networks, deeply participates in the dynamic balance of plaque stability. Its rhythmic disruption is not only a driving factor in disease progression but also a potential key target for early intervention. The proposal of time-sensitive microbiota intervention strategies marks the deep integration of microbiome medicine and chronobiology – by deciphering the interaction network of host-microbiota rhythms, integrating dietary interventions, microbiota regulation, and circadian synchronization into a multidimensional treatment system, providing a new dimension for cardiovascular disease prevention and treatment. However, the technical bottleneck of individualized rhythm adaptation and ecological safety challenges still urgently need to be broken through through innovative means such as multi-omics dynamic monitoring, AI-driven modeling, and adaptive clinical trials. In the future, with the in-depth deciphering of the trinity regulatory mechanism of “microbiota rhythm-host physiology-environmental cycle,” atherosclerosis prevention and treatment may enter the era of precision medicine in “spatio-temporal regulation,” initiating a profound transformation in chronic disease management from “correcting pathological abnormalities” to “rebuilding ecological rhythms.” Breakthroughs in this field will not only reshape the theoretical framework of cardiovascular medicine but also lead to cognitive upgrades in the life sciences regarding the dynamic balance of complex systems.

## Data Availability

Not applicable.
